# Supplementary material for: Revealing the neural fingerprints of a missing hand
Source: eLife. 2016 Aug 23;5:e15292. doi: 10.7554/eLife.15292 (PMC5040556; doi:10.7554/eLife.15292)
Supplement: Supplementary file 1. — DOI: http://dx.doi.org/10.7554/eLife.15292.014 [file elife-15292-supp1.docx]

|  | High field scans | | | | | Standard field scans | |
| --- | --- | --- | --- | --- | --- | --- | --- |
|  | Missing hand map | Missing hand overlap | Intact hand map | Intact hand overlap | Bimanual digit map | Bimanual digit map | Missing hand overlap |
| Above elbow | x | x | x | x | x | - | - |
| Below elbow | x | x | x | x | x | - | - |
| Brachial plexus | - | - | - | - | - | x | x |
| C1 | x | x | x | x | x | x | x |
| C2 | x | x | x | x | x | x | x |
| C3 | x | x | x | x | x | x | x |
| C4 | x | x | x | x | x | x | x |
| C5 | x | x | - | - | - | - | - |
| C6 | x | x | - | - | - | - | - |
| C7 | x | x | - | - | - | - | - |
| C8 | x | x | - | - | - | - | - |
| C9 | x | x | - | - | - | - | - |
| C10 | x | x | - | - | - | - | - |
| C11 | x | x | - | - | - | - | - |

Supplementary file 1. Runs acquired for each participant. x = acquired; - = not acquired.
